# Refractory splenic bleeding from splenic angiosarcoma: A case report and literature review

**DOI:** 10.1016/j.ijscr.2022.107708

**Published:** 2022-09-27

**Authors:** Tetsuro Kawazoe, Kippei Ohgaki, Eisuke Adachi, Yoichi Ikeda, Fumiyoshi Fushimi, Daisuke Kakihara

**Affiliations:** a Department of Gastrointestinal Surgery, Kyushu Central Hospital of the Mutual Aid Association of Public School Teachers, Fukuoka, Japan; b Department of Pathology, Kyushu Central Hospital of the Mutual Aid Association of Public School Teachers, Fukuoka, Japan; c Department of Radiology, Kyushu Central Hospital of the Mutual Aid Association of Public School Teachers, Fukuoka, Japan

**Keywords:** Case report, Atraumatic splenic rupture, Splenic angiosarcoma, Splenectomy

## Abstract

**Introduction and importance:**

Atraumatic splenic rupture (ASR) is a rare state that accounts for only <1 % of splenic ruptures. One of the causes of ASR is splenic neoplasm such as angiosarcoma. The treatment strategy for ASR is still unclear given the small number of cases reported in detail.

**Case presentation:**

A 75-year-old woman presenting with abdominal pain with shock was referred to our hospital. Emergency computed tomography revealed splenic rupture, and hemodynamic stabilization was obtained by emergent vascular embolization. Rebleeding occurred 27 days after the initial treatment, and splenectomy was performed. Pathologically, ASR was diagnosed as caused by splenic angiosarcoma.

**Clinical discussion:**

ASR is a very rare disease. The etiology of ASR has been reported as neoplastic, infectious, and so on. The treatment for ASR should be decided considering the etiology of ASR, hemodynamic stability, volume of blood transfusion, patient status, severity of the splenic injury, and volume of intraperitoneal bleeding.

**Conclusions:**

We experienced a very rare case of ASR, in which diagnosis was challenging and the timing of surgery was difficult to determine. When splenic rupture has an atraumatic cause, splenectomy should be considered because of the possibility of malignancy.

## Highlights

• The treatment strategy for traumatic splenic rupture is conservative therapy if possible, but that for atraumatic splenic ruptures has not been elucidated.
• In the case of conservative treatment for atraumatic splenic rupture, pathological examination cannot be performed, which may leave the patient untouched if the disease is malignant and at risk of rebleeding.
• Given the challenging diagnostics of angiosarcoma, splenectomy should be considered early in cases of atraumatic splenic rupture.

## 1 Introduction

Splenic rupture is divided into traumatic and atraumatic cases. Atraumatic splenic rupture (ASR) is extremely rare, accounting for <1 % of splenic rupture. Causes of ASR included neoplasms, infections, inflammation, drugs, and others [1]. Immediate diagnosis and early treatment are necessary to ensure patient survival and improve the clinical course.

The principal treatment strategy for traumatic splenic hemorrhage is conservative therapy without surgery, if possible [2]. However, the treatment strategy for ASR has not been established. Herein, we describe a rare case of splenic angiosarcoma causing ASR that required emergent embolization followed by splenectomy and provide a brief review of literature. Our work has been reported in line with the SCARE Guidelines [3].

Case Presentation.

**Fig. 1:**
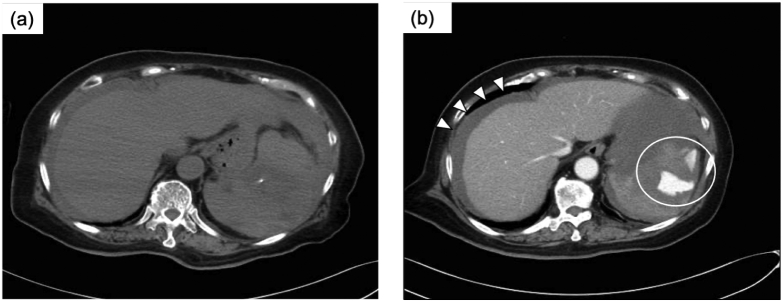
CT of the abdomen on admission Simple CT (a) and contrasted CT (b) of the abdomen showed hematogenous ascites effusion (arrowhead) not only around the liver and spleen but also in the pelvis and extravasation from the cranial side of the spleen implicating active bleeding (white circle). CT, computed tomography.

A 75-year-old woman presenting with sudden upper abdominal pain was transported to our emergency department soon after the onset. She had no history of trauma. She had a past medical history of atrial fibrillation that required anticoagulation therapy with apixaban. She did not have specific family history and psychiatric history. Her level of consciousness was clear, but her blood pressure decreased to 70/40 mmHg. Her pulse rate was 85 beats per minute. Emergent contrasted computed tomography (CT) of the abdomen showed hematogenous ascites effusion not only around the liver and spleen but also in the pelvis and extravasation from the cranial side of the spleen implicating active bleeding (Fig. 1).

Laboratory tests showed a white blood cell count of 7200/μL (normal range, 3300–8600/μL), red blood cell count of 3.30 × 10^6^/μL (normal range, 3.86–4.92 × 10^6^/μL), hemoglobin of 8.7 g/dL (normal range, 11.6–14.8 g/dL), platelet count of 56 × 10^3^/μL (normal range, 158–348 × 10^3^/μL), and prothrombin activity of 66.7 % (normal range, 80 %–120 %). Blood gas analysis showed lactate level of 35 mg/dL (normal range, 4.5–14.4 mg/dL). The initial diagnosis was hypovolemic shock due to splenic bleeding, followed by ASR, which required emergent interventional surgery laboratory and imaging survey.

Under local anesthesia, the right femoral artery was punctured, and a 4-Fr catheter was inserted in the right femoral artery. Celiac arteriography and selective splenic arteriography demonstrated contrast extravasation from the superior branch of the splenic artery. Transcatheter embolization using gelatin sponge particles and metallic coil was performed in the superior branch of the splenic artery. Finally, selective splenic arteriography revealed no contrast extravasation.

After the emergent vascular embolization, her blood pressure stabilized to 120/72 mmHg. Our treatment plan was to initiate a search for the cause of ASR and perform an elective surgery, if necessary. The serum levels of tumor markers such as carcinoembryonic antigen (2.0 ng/mL; normal range, <5.0 ng/mL) and carbohydrate antigen 19–9 (22.7 U/mL; normal range, <37 U/mL) were within the normal range, whereas the serum level of soluble interleukin-2 receptor was slightly increased (622 U/mL; normal range, 122–496 U/mL). Tests for cytomegalovirus and antinuclear antibodies were negative. As the cause of ASR could not be identified, we planned to perform elective splenectomy. However, as the platelet count gradually decreased during the clinical course (18 × 10^3^/μL on the 23rd hospital day), it was difficult to determine the timing of elective surgery.

**Fig. 2:**
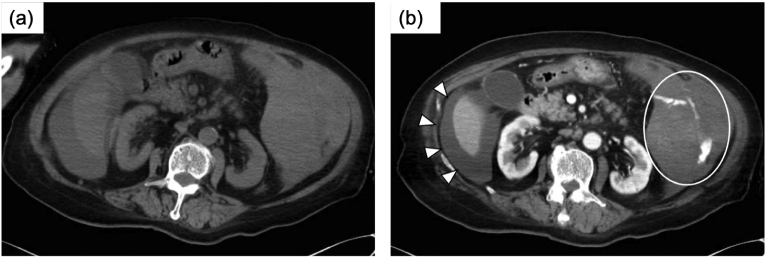
CT of the abdomen on the 28th hospital day Simple CT (a) and contrasted CT (b) of the abdomen showed hematogenous ascites effusion (arrowhead) and extravasation from the caudal side of the spleen implicating active bleeding from the inferior branch of the splenic artery (white circle).

On the 28th hospital day, her level of consciousness fell into Japan Coma Scale 100 and her blood pressure dropped to 70/45 mmHg. Emergent contrasted CT of the abdomen showed hematogenous ascites effusion and extravasation from the caudal of the spleen implicating active bleeding from the inferior branch of the splenic artery (Fig. 2).

**Fig. 3:**
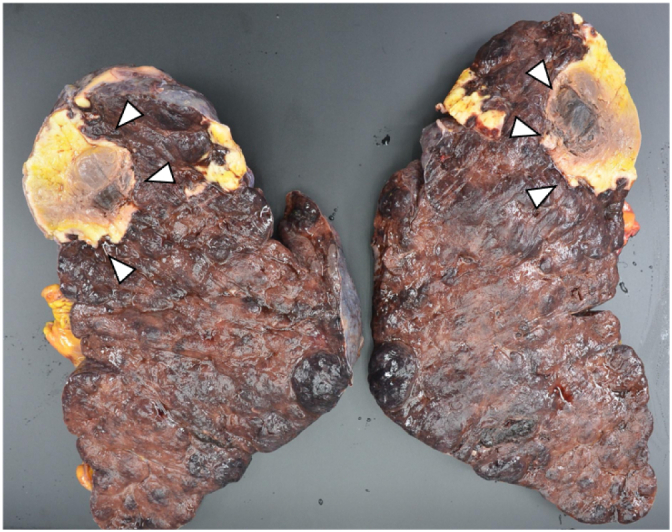
Resected specimen of the spleen The spleen was markedly enlarged, and the size was 22 × 13 × 10 cm. Necrotic site (arrowhead), with a size of 6.0 × 6.0 cm, was observed in the upper pole of the spleen.

Emergent splenectomy was performed on the 28th hospital day by TK (a fully trained surgeon). Hemorrhage from the splenic artery was observed. The operation time was 77 min, and the intraoperative blood loss was 4034 mL. The spleen was markedly enlarged, and the size was 22 × 13 × 10 cm. A necrotic site, with a size of 6.0 × 6.0 cm, was observed in the upper pole of the spleen (Fig. 3). The postoperative course was uneventful, and she was discharged on the 25th postoperative day.

**Fig. 4:**
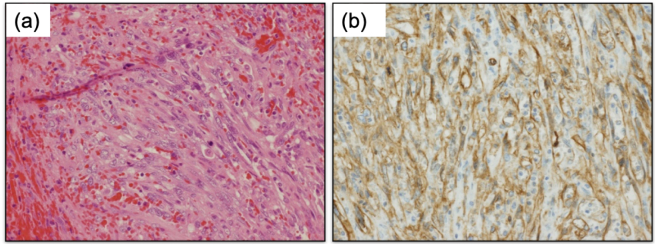
Histological findings of splenic angiosarcoma Pathological examination showed proliferation of oval- to spindle-shaped cells having hyperchromatic nuclei arranged in an irregular microtubular pattern, accompanied by massive hemorrhage and necrosis (a, ×200). Immunohistochemically, the tumor cells were positive for CD34 (b, ×200).

Pathological examination of the resected specimen demonstrated proliferation of oval- to spindle-shaped cells having hyperchromatic nuclei arranged in an irregular microtubular pattern, accompanied by massive hemorrhage and necrosis. Intracytoplasmic vacuoles were also noted. Mitotic figures were frequently seen (5/10 high-power field). Immunohistochemically, the tumor cells were positive for CD31, CD34, ERG, CD4, CD8 (focal), CD68 (focal), CD163, and p16 but negative for D2-40, HHV-8, C-MYC, and p53. The Ki-67 labeling index was 30 % (Fig. 4). The findings were suggestive of splenic vascular neoplasms such as splenic angiosarcoma or angioma, but we could not determine whether the nature of the tumor was benign or malignant at that time.

At the 7-month follow-up visit after the surgery, contrasted CT of the abdomen showed multiple low-dense masses in the liver indicating liver metastases. From the pathological findings and clinical course, we diagnosed the tumor as splenic angiosarcoma.

## 2 Discussion

ASR, splenic rupture without trauma, is a very rare disease. The etiology of ASR has been reported as neoplastic, infectious, inflammatory noninfectious, drug- and treatment-related, and mechanical, with a normal spleen, and the overall ASR-related mortality rate was 12.2 % [4]. The most common causes of ASR are hematological malignancies such as acute leukemia and viral infection such as cytomegalovirus infection [4].

The treatment for ASR should be decided considering the etiology of ASR, hemodynamic stability, volume of blood transfusion, patient status, severity of the splenic injury, and volume of intraperitoneal bleeding [5]. Among the reported ASR cases, 85.3 % of the patients had surgery within 24 h of the diagnosis of splenic rupture and 2.1 % of the patients underwent secondary splenectomy in the event of rebleeding and hemodynamic instability [4]. Interventional radiological therapy has the advantage of preserving normal spleen tissue and its immunological function in infectious etiology. On the contrary, in the case of conservative treatment, pathological examination cannot be performed, which may leave the patient untouched if the disease is malignant and at risk of rebleeding.

Angiosarcoma is quite a rare malignant tumor originating from the endothelial cells, and its main loci are the skin (33 %), soft tissues (24 %), breast (8 %), bone marrow (6 %), and spleen (4 %). Regardless of the organ of primary focus in such a disease, it is difficult to prevent rapid metastases [6]. Concerning about primary splenic angiosarcoma, the majority of patients presented with abdominal pain that was most frequently localized to the left upper quadrant. Fatigue, fever, and weight loss are also common symptoms. Moreover, 13 % of the patients presented with ASR [7], and 14.3 %–32.5 % of patients with splenic angiosarcoma have been reported to present thrombocytopenia [7], [8]. The etiology of primary splenic angiosarcoma is unknown. Some authors believe that primary splenic angiosarcoma develops from previously existing benign tumors such as hemangiomas and lymphangiomas [9], [10]. Thorium dioxide and vinyl chloride have been suggested to cause hepatic angiosarcoma, but there have been no etiologic associations between chemical agents and splenic angiosarcomas [11].

Regarding the treatment of ASR, total splenectomy has been selected in most cases. Organ-sparing surgery such as partial splenectomy and conservative treatment has also been reported [4], [12]. Although embolization may be useful in stabilizing hemodynamics, splenectomy should be considered if the cause of bleeding is neoplastic. The prognosis of splenic angiosarcoma is very poor, with an average survival time of approximately 8 months. Tumor size of >5 cm and splenic rupture have been reported as prognostic markers of splenic angiosarcoma [13].

In the present case, we performed vascular embolization immediately after the diagnosis of hypovolemic shock due to ASR and obtained hemodynamic stabilization. We attempted to identify the cause of ASR, but we were unable to identify the cause and planned an elective splenectomy. An earlier splenectomy might have been attempted to determine the cause, but the low platelet count during the clinical course made it difficult to determine the timing of the surgery. Because the patient developed rebleeding from the spleen, we performed an emergent splenectomy.

Kornmann et al. reported a case of splenic angiosarcoma that presented rebleeding 17 months after vascular embolization [14]. Tataria et al. reported a case of splenic angiosarcoma that developed rebleeding 15 days after the laceration of the spleen [12]. In our case, the period from the first embolization to rebleeding was 27 days. From these findings, splenectomy should be considered even if hemodynamic stabilization is obtained by conservative therapy in the case of ASR that was thought to be caused by splenic angiosarcoma.

## 3 Conclusions

We have demonstrated a very rare case of ASR, in which diagnosis was challenging and determining the timing of surgery was difficult. Although we successfully achieved hemodynamic stabilization, the patient developed rebleeding 27 days after vascular embolization. This case demonstrates the importance of the consideration of early surgical intervention in ASR even after the achievement of hemodynamic stabilization. Given the challenging diagnostics of angiosarcoma, splenectomy should be considered early in cases of ASR with even the slightest suspicion of angiosarcoma, such as thrombocytopenia.

## Abbreviations

ASR: atraumatic splenic rupture
CT: computed tomography

## Sources of funding

This study is supported by 10.13039/501100001691JSPS KAKENHI (JP 22K16526).

## Ethical approval

This study was conducted in accordance with the ethical standards of our institution.

## Consent for publication

Written informed consent was obtained from the patient for the publication of this case report and any accompanying images.

## Research registration

N/A.

## Guarantor

Tetsuro Kawazoe

## Provenance and peer review

Not commissioned, externally peer-reviewed.

## CRediT authorship contribution statement

TK conceived the case presentation and drafted the manuscript. KO, EA, DK, and YI participated in the treatment of the patient. FF determined the pathological diagnosis of the patient. All authors read and approved the final manuscript.

## Declaration of competing interest

The authors declare no conflict of interest in this paper.
